# A virtually supervised exercise program improved fitness and mental wellness in healthy and comorbidity older adult individuals during the COVID-19 pandemic

**DOI:** 10.3389/fpubh.2024.1328518

**Published:** 2024-04-23

**Authors:** Ermilo Canton-Martínez, Iván Rentería, Juan Pablo Machado-Parra, Rubén Avilés Reyes, José Moncada-Jiménez, David K. Johnson, Olga Molinero Gonzalez, Alfonso Salguero Del Valle, Alberto Jiménez-Maldonado

**Affiliations:** ^1^Facultad de Deportes, Universidad Autónoma de Baja California, Ensenada, Mexico; ^**2**^Institute of Biomedicine (IBIOMED) and Department of Physical Education, University of León, Campus Universitario, León, Spain; ^3^Facultad Ciencias Administrativas y Sociales Universidad Autónoma de Baja California, Ensenada, Mexico; ^4^Human Movement Sciences Research Center (CIMOHU), University of Costa Rica, San José, Costa Rica; ^5^Department of Neurology, University of California, Davis, Davis, CA, United States

**Keywords:** older adults, physical exercise, wellness, fitness level, social distancing

## Abstract

**Background:**

The COVID-19 pandemic affected older adults worldwide. Sedentary older adults experienced more severe adverse health effects due to their shelter-in-place. Physical activity was strongly recommended during periods of social distancing. The present study evaluated the impact of a virtually supervised exercise program on the physical fitness and mental health of Mexican older adults during the pandemic’s lockdown.

**Methods:**

Participants were 44 older adults who were assigned to one of four physical fitness groups: a healthy control group (Ctrl-H, *n* = 15), a comorbidity control group (Ctrl-COM, *n* = 9), an exercise group without comorbidities (Exe-H, *n* = 11), and an exercise group with comorbidities (Exe-COM, *n* = 9). The participants engaged in a 60-min, virtually-supervised concurrent exercise session three times/week for 12 weeks. Fitness was measured using the online Senior Fitness Tests and the 4-m Gait Speed Test. Mental health was evaluated through virtual interviews using the Hamilton Depression Rating Scale, the Geriatric Depression Scale, and the Connor-Davidson Resilience Scale. Within-subject pre vs. post-intervention comparisons tested for significant differences, between-groups and over time.

**Results:**

Significant interactions were found in the scores of the Geriatric Depression Scale (*p* ≤ 0.0001; η_p_^2^ = 0.35), the Hamilton Depression Scale (*p* ≤ 0.0001; η_p_^2^ = 0.35), resilience scores (*p* ≤ 0.0001; η_p_^2^ = 0.46), lower-body strength (*p* ≤ 0.0001; η_p_^2^ = 0.32), timed up-and-go test (*p* = 0.018; η_p_^2^ = 0.18), the 6MWT distance scores (*p* ≤ 0.0001; η_p_^2^ = 0.39), and the 4-m gait speed test scores (*p* = 0.011; η_p_^2^ = 0.20).

**Conclusion:**

A long-term virtually-supervised exercise program conducted during the COVID-19 lockdown period led to marked improvements in both the fitness and mental health of older Mexican adults. Comorbidities did not diminish these benefits. These findings provide empirical support for online exercise programs in the daily routines of older adults to make clinically meaningful improvements in both physical and mental well-being.

## Introduction

SARS-CoV-2 (Severe Acute Respiratory Syndrome Coronavirus-2) spread worldwide quickly (1–3), with more severe SARS-CoV-2 cases reported more frequently in older adults and people with chronic diseases (hypertension, type 2 diabetes, cardiovascular diseases, and comorbidity patients) (4).

COVID-19 was a whole-body infection that affected the brain, heart, insulin regulation, and other organs, thus exacerbating chronic disease (5, 6). Health systems around the world implemented preventive medicine strategies that included widespread shelter-in-place commands, quarantine, and limiting participation in outdoor activities, including sports, and exercise. Public gatherings and travel were severely curtailed. During that time, many public health officials highlighted that social distancing and isolation would also induce an inactive and sedentary behavior lifestyle, thus having deleterious downstream effects on physical fitness and mental health (2, 7, 8). Moreover, it is well known that the social distancing impact negatively on the life quality (including emotional health), in older adults with comorbidities (9, 10, 11), additionally authors have previously identified and suggested bidirectional association among diabetes, and cardiovascular diseases with mood disorders in adults and older adults (12, 13). On the other hand, in older adults, the sedentary behavior facilitates body weight gain, harms the function of the cardiovascular system, impairs the immune system responses, and increases the risk of suffering psychological and mental disorders (e.g., depression, psychological stress, and anxiety) and low resilience (14–16). Likewise, authors have previously identified that the sedentary behavior is a strong independent risk factor to suffer cardiovascular diseases in older adults with diabetes (17). The previous information emphasizes the need to practice physical activity in healthy and commorbidiy older adults, this kind of intervention was widely recommended throughout social distancing (9, 18–23), the recommendations included practice aerobic exercise (e.g., walking) complemented by balance and strength exercises (21, 23). Despite the widespread implementation of these recommendations, there is scant empirical data on the long-term impact of PE sessions using virtual supervision.

The current study assessed the impact of a virtually-supervised exercise training program during COVID-19 quarantine. We collected outcomes data change in physical fitness and mental health in healthy Mexican older adult controls compared to matched patients with comorbidity chronic disease. We hypothesized that a home-based exercise program, supported by virtual supervision, would induce positive physical fitness and mental health outcomes in all individuals; however, older adults with comorbid chronic disease would benefit most.

## Methods

### Study design and sample size

The current project followed a pragmatic clinical trial (non-random assignment) performed using virtual tools such as Facebook, WhatsApp, and Google Meet. The sample size comprised 44 older adults. First, the researchers advertised the project through a Facebook social media message. The interested people attended a video virtual meeting (Google Meet) to receive detailed information about the procedures and the aim of the study. Second, all interested individuals signed an informed consent (Google Forms). We assigned older adults to a *control group* (total sample = 24) subdivided into two groups: *Healthy control* (Ctrl-H, *n* = 15) individuals with no reported disease, and a *Comorbidity control* (Ctrl-COM, *n* = 9) group that included participants with self-reported hypertension, diabetes, or hypertension/diabetes. Both groups did not attend the training program; they were allowed to practice PE according to their lifestyle. Third, a *training group* (total sample = 20), included two groups: *Exercise-Healthy* (Exe-H, *n* = 11) individuals (no self-reported comorbid disease) and *Exercise comorbidity* (Exe-COM, *n* = 9) who reported hypertension, diabetes, or hypertension/diabetes. The exercise groups attended a combined concurrent exercise training program (12 weeks, three times per week) with virtual supervision. The study was completed between February and April 2021 and the protocol was approved by the Research Ethics Committee of the Facultad de Medicina y Psicología Campus Tijuana de la Universidad Autónoma de Baja California, México. The protocol was registered under the code 889/2020–2.

### Cognitive testing, depression and resilience measurements

**Figure 1:**
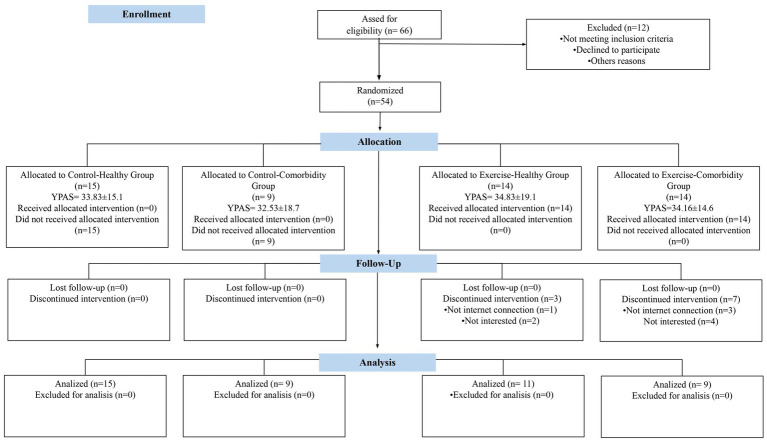
Flow diagram. Ctrl-H, Control Healthy Group; Ctrl-COM, Control Comorbidity Group; Exe-H, Exercise-Healthy Group; Exe-COM, Exercise Comorbidity Group; HDRS, Hamilton Depression Scale; GDS, Geriatric Depression Scale; MMSE, Mini Mental State Exam; CD-RISC, Connor Davidson Resilience Scale, YPAS, Yale Physical Activity Survey (h·wk. − 1).

Questionnaire completion was supported by staff trained in Google Forms. The depression and anxiety symptom inventories completed by the individuals were the *Hamilton Depression Scale* of 17 items (HDRS) (24). The HDRS was previously identified as a tool with good internal consistency (Cronbach’s alpha = 0.789) (25), and the *Geriatric Depression Scale* 15 items (GDS-15) (26, 27), the GDS is a valid method to assessing depressive symptoms in Hispanic and Latin American population, having cutoff of 4 to define a depressive condition in the individuals (28). The global cognition was assessed by the *Mini-Mental State Examination* [MMSE; Cronbach alpha for Mexican-American (Spanish Interview) = 0.83] (29). The HDRS, GDS, and MMSE are neuropsychological tools used in Mexican older adults (30). Finally, resilience was measured by the *Connor Davidson Resilience Scale* (CD-RISC) (31), an instrument used in the exercise training intervention (32), and validated in Spanish language (Cronbach’s alpha = 0.79) (33, 34). The cut off score for CD-RISC has not been established yet, however, it is well known that a higher CD-RISC score reflects a high resilience level (31). Each questionnaire was supported by staff trained in Google Forms. During the questionnaire design, the researchers team made several trials about the hyperlink of neuropsychological testing, and answered those, checking if the collected information was saved in a particular database (Excel file). Once revised that hyperlink of neuropsychological testing, and the data base was correctly, the researchers shared the questionnaire to the participants who were supervised by trained staff using a synchronous virtual meeting strategy. The study’s design is presented in Figure 1.

### Physical fitness assessment

Remote assessments (through Google Meet) physical fitness was assessed with the *Senior Fitness Test* (SFT) (35), for this, we followed the methodology previously published for online testing (36–38). In brief, the webcam was positioned in front of each participant during the test. The SFT battery was demonstrated by a licensed instructor in practice, and the participants were asked to repeat the exercises. In order for the exercises to be perfectly performed, the corrections were performed by the instructor when necessary. The physical fitness tests were performed following this order:

*The 30-s chair test* consisted of performing the maximum number of standing movements for 30 s from and sitting in a chair of 45 cm height approximately to measure lower-body strength. *The Curl Up Test:* Consisted in perform the maximum number of biceps curl-ups using a water bottle (2.25 kg for women and 2.0 kg for men) during 30 s to measure upper-body strength. *The 8-foot timed up-and-go Test* (TUG): was used to measure dynamic balance the participants were seated in a chair. A bottle of water was placed 2.44 m in front of the front edge of the chair. The participant walked as soon as possible through the bottle of water to get back to the chair and the time was recorded. *The* timed *4-meter Gait Speed Test*: was recorded is a test considered a good measure of disability and a predictor of poor clinical outcomes in older adults, the static 4 meter test was performed in the backyard or front yard, or on the sidewalk to have the space necessary to perform (39, 40). To carry out the test, a flat route free of obstacles was identified, and 4 m were marked with adhesive tape. The participant stood with his toes touching the starting line. Subsequently standardized instructions were given: “walk to the line marked by the adhesive tape, walk in your usual gait speed and come to a sudden stop when yo have reached the cone ready … start.” Stopwatch timing began when the participant began to move. Timing stopped when the first participant’s foot completely crossed the 4 m line (41, 42). Members of the research team (name’s initials will be disclosed following peer review) supervised and trained staff using a synchronous virtual meeting strategy. Additionally, at the begin of the study, the Yale Physical Activity Survey (YPAS) was used to determine the physical activity levels in the participants. The YPAS is a questionnaire validated in Spanish language (43).

### Exercise protocol

All virtual exercise sessions were supervised by researchers and trained staff through Google Meet video calls. Each session lasted 60 min and consisted of a warming-up (10 min), a main workout (40 min), and a cool-down (10 min). During warm-up, passive stretching, and mobility exercises were performed. The main workout included basic aerobic exercises such as walking inside the house twice (5 min walk and 1 min rest), five times high knees (30 s and 30-s rest), five times side steps (30 s and 30-s rest), and five times leg curl (30 s and 30-s rest).

The strength exercises involved a chair stand, biceps curls, one-arm row, and triceps extensions using water bottles (1–2 L), wall push-ups, leg extensions, standing heel raises, standing kickbacks, and abdominal crunches. For the cool-down, passive stretching exercises were performed. All the exercises were performed at moderate intensity (i.e., *Borg’s rating of perceived exertion Scale* from 6 to 20) where the participants had to reach an intensity between 12 and 14 points (44), for the last, the researchers made adjustment in the number of repetitions.

### Statistical analysis

Descriptive statistics are presented as the mean and standard deviation (M ± SD) unless otherwise noted. The intention-to-treat analysis approach was used in this study, given the drop-out of participants in the exercise groups (3 in the healthy and 7 in the comorbidity group). According to Little (45), we determined that missing data were generated completely at random (MCAR). Thus, we first used the mean imputation method, where the dependent variable of interest’s arithmetic mean in the post-test scores recorded was imputed to the post-test participant’s missing values (46). Then, we computed inferential statistics, including 4 × 2 *mixed ANOVA tests* (4 groups x 2 measurements) for physical and cognitive variables. Third, we conducted a sensitivity analysis using the median imputation method to assess the robustness and reliability of the results. The *Least Difference Significant (LSD) post hoc* test followed significant ANOVA interactions, and the 95% confidence intervals (CI95%) for the mean differences are presented. Finally, the *effect size* (ES) was estimated by *partial* η_p_^2^ and *Cohen’s d*. The η_p_^2^ ES was interpreted as *trivial* (0.01–0.059), *medium* (0.06–0.139), and *large* (≥ 0.14), and Cohen’s *d* as *trivial* (< 0.2–0.49), *medium* (0.5–0.75), and *large* (≥ 0.80) (47). The overall statistical significance was set *a priori* at *p* < 0.05. Statistical analyses were performed with the IBM-SPSS program (IBM Corp., Armonk, NY, United States), version 26.0.

## Results

### Baseline characteristics

Descriptive statistics for the participants using two methods of imputation for the intention-to-treat analysis are sown in Table 1. The sensitivity analysis is shown by using different imputation scenarios and ANOVA interactions to assess the robustness of results. ANOVA interaction *p*-values were similar in all but one variable (i.e., upper-body strength) when using the mean and the median imputation.

**Table 1:** Descriptive statistics using two methods of imputation for the intention-to-treat analysis.

			Imputation method					
			Arithmetic mean imputation			Median imputation		
Group/variable	Pre-test	Post-test	A×B (*p* ≤)	A (*p* ≤)	B (*p* ≤)	A×B (*p* ≤)	A (*p* ≤)	B (*p* ≤)
Comorbid control (*n* = 9)								
Lower-body strength (reps)	15.11 ± 4.37	13.22 ± 4.02	0.0001	0.979	0.011	0.0001	0.981	0.012
Upper-body strength (reps)	18.67 ± 3.87	21.11 ± 5.78	0.099	0.0001	0.521	0.036	0.0001	0.476
Trunk flexibility (cm)	−1.56 ± 1.24	−2.78 ± 3.03	0.454	0.934	0.035	0.280	0.662	0.023
Arm flexibility (cm)	−4.11 ± 5.30	−6.00 ± 5.83	0.121	0.085	0.008	0.154	0.115	0.011
Timed up-and-go (s)	7.10 ± 2.48	8.06 ± 2.65	0.018	0.572	0.019	0.012	0.661	0.016
6MWT (m)	634.33 ± 73.76	619.00 ± 67.97	0.0001	0.001	0.011	0.0001	0.001	0.011
4-m gait speed (s)	7.17 ± 1.51	7.28 ± 1.66	0.011	0.122	0.153	0.002	0.041	0.151
Geriatric depression scale (pts.)	5.56 ± 1.74	4.67 ± 1.58	0.0001	0.0001	0.001	0.0001	0.0001	0.001
Hamilton Depression Scale (pts.)	8.89 ± 3.82	9.78 ± 3.99	0.0001	0.008	0.043	0.0001	0.004	0.036
Resilience (pts.)	27.22 ± 5.22	26.67 ± 4.61	0.0001	0.0001	0.009	0.0001	0.0001	0.008
Healthy control (*n* = 15)								
Lower-body strength (reps)	15.47 ± 4.55	14.40 ± 3.91						
Upper-body strength (reps)	17.67 ± 4.42	18.60 ± 3.18						
Trunk flexibility (cm)	−3.20 ± 3.78	−3.27 ± 3.62						
Arm flexibility (cm)	−2.60 ± 4.64	−0.67 ± 6.25						
Timed up-and-go (s)	7.98 ± 2.13	8.44 ± 2.33						
6MWT (m)	648.20 ± 42.18	620.53 ± 60.51						
4-m gait speed (s)	6.06 ± 1.11	6.57 ± 0.92						
Geriatric depression scale (pts.)	4.73 ± 1.53	4.80 ± 1.78						
Hamilton Depression Scale (pts.)	7.33 ± 1.76	7.27 ± 2.49						
Resilience (pts.)	28.20 ± 4.30	28.60 ± 3.78						
Comorbid experimental (*n* = 12)								
Lower-body strength (reps)	17.92 ± 3.70	17.43 ± 2.63						
Upper-body strength (reps)	16.75 ± 3.91	20.32 ± 2.30						
Trunk flexibility (cm)	−1.25 ± 2.67	−0.43 ± 0.87						
Arm flexibility (cm)	−3.27 ± 6.51	−6.99 ± 6.73						
Timed up-and-go (s)	6.51 ± 1.31	6.28 ± 1.23						
6MWT (m)	649.42 ± 110.61	744.22 ± 38.90						
4-m gait speed (s)	6.38 ± 1.76	5.43 ± 1.68						
Geriatric depression scale (pts.)	4.17 ± 1.90	1.33 ± 1.13						
Hamilton Depression Scale (pts.)	6.58 ± 3.06	5.57 ± 1.91						
Resilience (pts.)	29.17 ± 4.73	33.78 ± 2.33						
Healthy experimental (*n* = 18)								
Lower-body strength (reps)	15.89 ± 3.07	19.38 ± 2.57						
Upper-body strength (reps)	17.50 ± 3.50	18.47 ± 1.95						
Trunk flexibility (cm)	−2.00 ± 2.14	−1.38 ± 1.89						
Arm flexibility (cm)	−5.64 ± 4.49	−8.69 ± 5.01						
Timed up-and-go (s)	6.83 ± 1.91	6.09 ± 1.01						
6MWT (m)	641.00 ± 103.85	732.19 ± 49.92						
4-m gait speed (s)	7.02 ± 1.64	6.17 ± 1.52						
Geriatric depression scale (pts.)	5.06 ± 2.60	2.00 ± 1.33						
Hamilton Depression Scale (pts.)	8.11 ± 4.64	4.38 ± 2.23						
Resilience (pts.)	27.28 ± 4.66	34.62 ± 2.01						

ANOVA terms: AxB (Interaction between measurements and groups), A, Measurements (pre-test vs. post-test); B, Groups (Comorbid control, Healthy control, Comorbid experimental, Healthy experimental).

### Psychological profile

**Figure 2:**
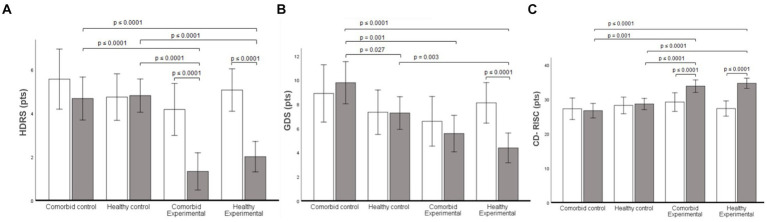
Mental health results for all groups. **(A)** Hamilton Depression Scale (HDRS) score of 17 items. **(B)** Geriatric Depression Scale (GDS) score. **(C)** Connor Davidson Resilience Scale (CD-RISC) score. Data are presented as mean. Error bars are the 95% confidence interval.

No significant statistical group by measurements interaction was found the Mini-Mental State Examination (*p* = 0.162; η_p_^2^ = 0.10). A statistically significant group by measurements interaction was found in the scores of the Geriatric Depression Scale-GDS score (*p* ≤ 0.0001; η_p_^2^ = 0.35). Follow-up analysis showed a reduction in depression scores in the Exe-COM group (*p* ≤ 0.0001, CI95% = −1.72, −3.97 pts.), and the Exe-H group (*p* ≤ 0.0001, CI95% = −2.14, −3.98 pts.). There were significant mean differences in the post-test scores between the Ctrl-COM and the Exe-COM group (*p* ≤ 0.0001, CI95% = 2.04, 4.65 pts.), the Ctrl-COM, and the Exe-H group (p ≤ 0.0001, CI95% = 1.46, 3.87 pts.), the Ctrl-H and Exe-COM group (*p* ≤ 0.0001, CI95% = 2.22, 4.62 pts.), and the Ctrl-H and Exe-H group (p ≤ 0.0001, CI95% = 1.77, 3.83 pts.; Figure 2A). A statistically significant group by measurements interaction was found in the scores of the Hamilton Depression Scale (*p* ≤ 0.0001; η_p_^2^ = 0.35). Follow-up analysis showed a reduction in depression scores in the Exe-H group (*p* ≤ 0.0001, CI95% = −2.53, −4.94 pts.). There were significant mean differences in the post-test scores between the Ctrl-COM and the Ctrl-H group (*p* = 0.027, CI95% = 0.31, 4.72 pts.), Ctrl-COM and the Exe-COM group (*p* = 0.001, CI95% = 1.90, 6.52 pts.), the Ctrl-COM and the Exe-H group (*p* ≤ 0.0001, CI95% = 3.26, 7.54 pts.), and the Ctrl-H and Exe-H group (*p* = 0.003, CI95% = 1.06, 4.72 pts.; Figure 2B). A statistically significant group by measurements interaction was found in the resilience scores (*p* ≤ 0.0001; η_p_^2^ = 0.46). Follow-up analysis showed an increased resilience in the Exe-COM group (*p* ≤ 0.0001, CI95% = 2.49, 6.75 pts.) and the Exe-H group (*p* ≤ 0.0001, CI95% = 5.61, 9.08 pts.). There were significant mean differences in the post-test scores between the Ctrl-COM and the Exe-COM group (*p* ≤ 0.0001, CI95% = −4.32, −9.91 pts.), the Ctrl-COM and the Exe-H group (*p* ≤ 0.0001, CI95% = −5.37, −10.54 pts.), the Ctrl-H and Exe-COM group (*p* ≤ 0.0001, CI95% = −2.73, −7.64 pts.), and the Ctrl-H and Exe-H group (*p* ≤ 0.0001, CI95% = −3.81, −8.24 pts.; Figure 2C).

### Physical parameters

**Figure 3:**
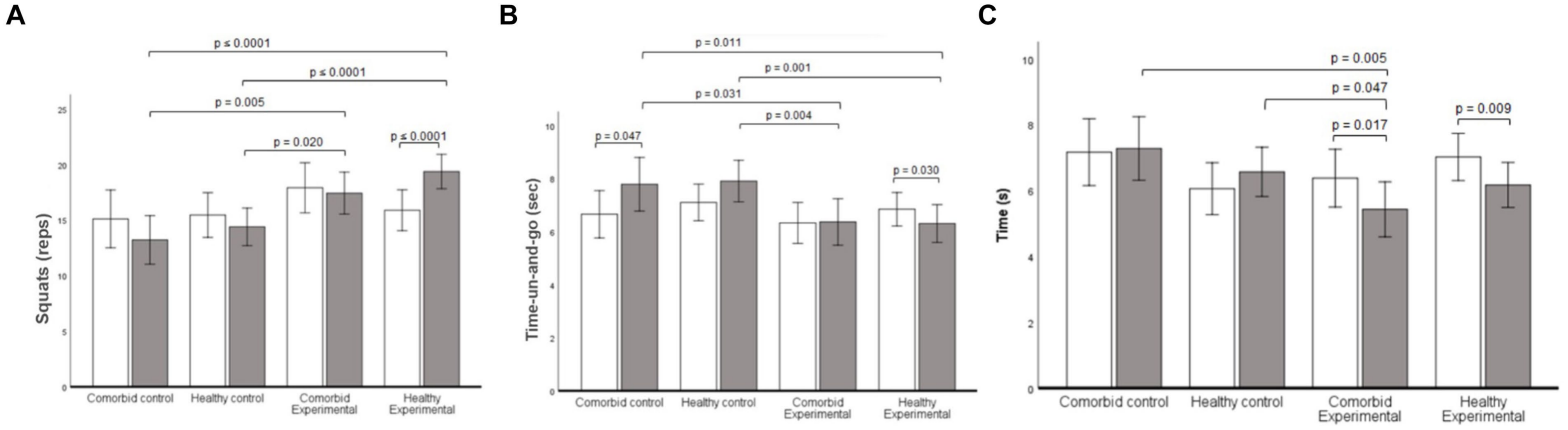
Fitness results throughout the program for all groups. **(A)** Squat repetitions in 30  sec. **(B)** Timed-Up-and-Go (TUG) in sec. **(C)** 4-meter gait speed test in sec. Data are presented as mean. Error bars are the 95% confidence interval.

No significant statistical group by measurements interaction was found on upper-body strength (*p* = 0.099; η_p_^2^ = 0.12), trunk flexibility (*p* = 0.454, η_p_^2^ = 0.05), and arm flexibility (*p* = 0.121, η_p_^2^ = 0.11). A statistically significant group by measurements interaction was found on lower-body strength (*p* ≤ 0.0001; η_p_^2^ = 0.32). Follow-up analysis showed an increase in lower-body strength only in the Exe-H group (*p* ≤ 0.0001, CI95% = 1.90, 5.08 reps.). There were significant mean differences in the post-test scores between the Ctrl-COM and the Exe-COM participants (*p* = 0.005, CI95% = −1.32, −7.10 reps.), the Ctrl-COM and the Exe-H participants (*p* ≤ 0.0001, CI95% = −3.48, −8.83 reps.), the Ctrl-H and Exe-COM participants (*p* = 0.020, CI95% = −0.50, −5.57 reps.), the Ctrl-H and Exe-H participants (*p* ≤ 0.0001, CI95% = − 2.69, −7.27 reps.; Figure 3A).

A statistically significant group by measurements interaction was found on the timed up-and-go test (*p* = 0.018; η_p_^2^ = 0.18). Follow-up analysis showed a reduction in time in the comorbid control (*p* = 0.047, CI95% = −0.01, −1.91 s) and Exe-H (*p* = 0.030, CI95% = −0.07, −1.42 s) groups. There were significant mean differences in the post-test scores between the Ctrl-COM and the Exe-COM participants (*p* = 0.031, CI95% = 0.17, 3.40 s), the Ctrl-COM and the Exe-H participants (*p* = 0.011, CI95% = 0.48, 3.47 s), the Ctrl-H and Exe-COM participants (*p* = 0.004, CI95% = 0.74, 3.58 s), and the Ctrl-H and Exe-H participants (*p* = 0.001, CI95% = 1.07, 3.63 s; Figure 3B). A statistically significant group by measurement interaction was found on the 4-m gait speed test scores (*p* = 0.011; η_p_^2^ = 0.20). Follow-up analysis showed faster speed times in the Exe-COM (*p* = 0.017, CI95% = −0.18, −1.72 s) and Exe-H (*p* = 0.009, CI95% = −0.22, −1.48 s) groups. There were significant mean differences in the post-test scores between the comorbid control and the Exe-COM participants (*p* = 0.005, CI95% = 0.57, 3.13 s), and the Ctrl-H and Exe-COM participants (*p* = 0.047, CI95% = 0.02, 2.26 s; Figure 3C).

## Discussion

The current study assessed the utility of a home-based training program to improve fitness and mental health in two groups of Mexican older adults (with and without comorbid conditions). Repeated measures of the SFT and depression symptom inventories indicated that 36 exercise sessions improved both fitness and well-being in older adults with comorbid conditions and those without. Our virtual exercise program increased the strength of the lower body, agility, and gait speed, and reduced depressive symptoms. A home-based exercise training with individual virtual supervision is a feasible intervention to reduce depressive symptoms and increase physical activity despite the pandemic’s far-reaching shelter-in-place orders. These data converge with the substantial review articles and perspectives about the recommendations to practice physical exercise during the COVID-19 lockdown to improve mental wellness (9).

Depression is prevalent in older adulthood (48, 49) and is a complex and persistent syndrome in old age (50, 51). A score above 4 points on the GDS instrument for Mexican older adults is considered the cut-off point to flag participants as potentially depressed (28, 30). In regard with it, the Exe-COM, and EXE-H groups showed a reduction in the GDS score, reporting lower than 4 (no depressive symptoms), additionally, the EXE-H showed in the HDRS_17_ a reduction of 4 points, considering it a modest but clinically meaningful (52), those data indicate that the exercise performed at home reduces significantly the depressive symptoms in older adults, a response that was more consistently observed in EXE-H group. Salguero et al. (48) reported similar results where depressive symptoms were reported significantly less often in more physically active participants. That was true for both community-dwelling and older adults in assisted living. The finding converges with many other studies that show that virtual supervised exercise programs reduce depression (32). Notably, we could not measure neurobiological mechanisms of the improvement of depressive symptoms as a function of increased PE. Low circulating BDNF is linked to increased depressive symptoms in older adults, especially those with low activity levels and clinical conditions (53). Further, elevated PE is associated with increased circulating BDNF levels (53–55); however, the association is not well-established, and future studies should focus on measuring BDNF signaling.

Resilience, defined as the ability to successfully respond to acute stress, trauma, or adversities (56–58), was also enhanced by our exercise program, regardless of health status (Figure 2C). Resilience is another complex index of mental health (57, 59) that involves the hypothalamic–pituitary–adrenal (HPA) axis activity. In highly-stressed individuals (i.e., low-resilience), HPA activity is significantly higher than in people who report moderate low stress (56). Chronic over-activation of HPA suppresses the immune system (60), leaving individuals more susceptible to chronic disease. Moreover, there is a consistent association between low resilience levels and a high prevalence of mental disorders of all types (58). Our results are compelling and important because simple exercise virtual exercise regimens could be an easy-to-implement and effective treatment to improve resilience levels in older adults who need the most help (15, 32, 61). Further, this repeated measures experimental design was stronger than the cross-sectional reports in the literature currently (15, 61, 62).

In the present study, lower-body strength was improved by the supervised exercise program, regardless of health status (Table 1). In older adults, leg muscle mass (22) and chair stand (36) improve following a remote virtually-supervised exercise program. Better lower-limb strength reduces fall risk (35) and preserves functional autonomy (22, 63). Thus, morphological adaptations elicited by the training sessions could explain the increased lower-body strength, especially the increased muscle mass (64) and hypertrophic responses (65). Concurrent training induces neuromuscular adaptations to improve muscle quality, resulting in significant improvements in the rate of force production (66). Convergent with this, chair stand test data, gait speed, and the TUG test also improved significantly after the exercise intervention. The gait speed has been previously considered a the “sixth vital sign” (39), this variable was identified as one independent risk factor for disability, cognitive impairment, falls, and depression (67, 68), consequently, authors have mentioned that a slow gait speed reflect micro-cerebrovascular disease, pathology of both cochlear and vestibular sense organs, white matter hyperintensities (WMHs) particularly involving the frontal lobe, and peripheral neuropathy (67–69). The previous data emphasize the usefulness of virtual exercise training aimed at improving the lower-body variables associated with functional autonomy, and potentially the preservation of the functions at specific brain functions in older adults, one adaptations that was not attenuated by chronic diseases such as hypertension and type 2 diabetes.

Despite promising findings linked with the functional autonomy, the current study has some limitations; first, we acknowledge that our sample size was small, and the participants’ sex was uneven among groups. Sex is a relevant factor in response to exercise training (70, 71) and could have been controlled for in exercise assignments instead of *post hoc* statistical controls that were used. Second, the remote approach used in this work, did not provide other specific information about the potential morphophysiological adaptations (body composition analysis, surface electromyography, and so on). to induce better physical performance in the lower body. Third, metabolic variables were not collected, which prevented us from identifying if biological variables like blood glucose levels, diabetes, blood pressure, and circulating BDNF were also changing with physical fitness and self-reported mental health after a remote supervised exercise program.

## Conclusion

The present study demonstrated that virtual home-based individual supervised physical activity is an effective strategy to improve fitness (lower-body strength), and mental wellness in older individuals with or without comorbidities. Supervised online physical activity tailored for older adults is a convenient and effective strategy that induces clinically meaningful change in fitness and mental health. By replacing standard exercise equipment with items commonly found at home, we were able to implement an exercise program for the homebound.

## Data availability statement

The raw data supporting the conclusions of this article will be made available by the authors, without undue reservation.

## Ethics statement

The studies involving humans were approved by Research Ethics Committee of the Facultad de Medicina y Psicología Campus Tijuana de la Universidad Autónoma de Baja California, México, the protocol was registered under the code 889/2020–2. The studies were conducted in accordance with the local legislation and institutional requirements. The participants provided their written informed consent to participate in this study. Written informed consent was obtained from the individual(s) for the publication of any potentially identifiable images or data included in this article.

## Author contributions

EC-M: Conceptualization, Writing – original draft. IR: Methodology, Writing – original draft, Writing – review & editing, Conceptualization. JM-P: Methodology, Writing – original draft. RR: Methodology, Writing – original draft. JM-J: Data curation, Formal analysis, Writing – review & editing. DJ: Data curation, Formal analysis, Methodology, Writing – review & editing. OG: Formal analysis, Writing – review & editing. AV: Data curation, Writing – review & editing. AJ-M: Conceptualization, Formal analysis, Funding acquisition, Methodology, Resources, Writing – original draft, Writing – review & editing.

## References

[1] Haverkamp BF Wiersma R Vertessen K van Ewijk H Oosterlaan J Hartman E. Effects of physical activity interventions on cognitive outcomes and academic performance in adolescents and young adults: a meta-analysis. J Sports Sci. (2020) 38:2637–60. doi: 10.1080/02640414.2020.1794763, PMID: 32783695

[2] Wackerhage H Everett R Krüger K Murgia M Simon P Gehlert S. Sport, exercise and covid-19, the disease caused by the SARS-CoV-2 coronavirus. Deutsche Zeitschrift Fur Sportmedizin. (2020) 71:E1–E11. doi: 10.5960/dzsm.2020.441, PMID: 37356971

[3] Wiersinga WJ Rhodes A Cheng AC Peacock SJ Prescott HC. Pathophysiology, transmission, diagnosis, and treatment of coronavirus disease 2019 (COVID-19): a review. JAMA-J American Medical Association. (2020) 324:782. doi: 10.1001/jama.2020.12839, PMID: 32648899

[4] Costa FF Rosário WR Farias ACR Guimarães R de Souza R Gondim SD. Metabolic syndrome and COVID-19: an update on the associated comorbidities and proposed therapies. Diabetes Metab Syndr Clin Res Rev. (2020) 14:809–14. doi: 10.1016/j.dsx.2020.06.016, PMID: 32540733 PMC7286828

[5] Baig AM Khaleeq A Ali U Syeda H. Evidence of the COVID-19 virus targeting the CNS: tissue distribution, host-virus interaction, and proposed neurotropic mechanisms. ACS Chem Neurosci. (2020) 11:995–8. doi: 10.1021/acschemneuro.0c00122, PMID: 32167747

[6] Kaushik P Kaushik M Parveen S Tabassum H Parvez S. Cross-talk between key players in patients with COVID-19 and ischemic stroke: a review on neurobiological insight of the pandemic. Mol Neurobiol. (2020) 57:4921–4928. 32813238 10.1007/s12035-020-02072-4 PMC7434850

[7] Ferreira MJ Irigoyen MC Consolim-Colombo F Saraiva JFK De Angelis K Vida fisicamente ativa como medida de enfrentamento ao COVID-19. Arq Bras Cardiol. (2020) 114:601–602. 32324841 10.36660/abc.20200235

[8] Maffetone PB Laursen PB. The perfect storm: coronavirus (Covid-19) pandemic meets overfat pandemic. Front Public Health. (2020) 8:135. doi: 10.3389/fpubh.2020.00135, PMID: 32391307 PMC7190793

[9] Sepúlveda-Loyola W Rodríguez-Sánchez I Pérez-Rodríguez P Ganz F Torralba R Oliveira DV. Impact of social isolation due to COVID-19 on health in older people: mental and physical effects and recommendations. J Nutr Health Aging. (2020) 24:938–47. doi: 10.1007/s12603-020-1500-7, PMID: 33155618 PMC7597423

[10] Douglas H Georgiou A Westbrook J. Social participation as an indicator of successful aging: an overview of concepts and their associations with health. Aust Health Rev. (2016) 41:455–62. doi: 10.1071/AH16038 27712611

[11] Loyola WS Camillo CA Torres CV Probst VS. Effects of an exercise model based on functional circuits in an older population with different levels of social participation. Geriatr Gerontol Int. (2018) 18:216–23. doi: 10.1111/ggi.13167 29034615

[12] Fenton WS Stover ES. Mood disorders: cardiovascular and diabetes comorbidity. Curr Opin Psychiatry. (2006) 19:421–7. doi: 10.1097/01.yco.0000228765.33356.9f, PMID: 16721175

[13] Golden SH Lazo M Carnethon M Bertoni AG Schreiner PJ Roux AVD. Examining a bidirectional association between depressive symptoms and diabetes. JAMA. (2008) 299:2751–9. doi: 10.1001/jama.299.23.2751, PMID: 18560002 PMC2648841

[14] Gualano MR Moro GL Voglino G Bert F Siliquini R. Effects of COVID-19 lockdown on mental health and sleep disturbances in Italy. Int J Environ Res Public Health. (2020) 17:1–13. doi: 10.3390/ijerph17134779 PMC7369943 32630821

[15] Lancaster MR Callaghan P. The Effect of exercise on resilience, its mediators and moderators, in a general population during the UK COVID-19 pandemic in 2020: a cross-sectional online study. BMC Public Health. (2022) 22:827. doi: 10.1186/s12889-022-13070-7, PMID: 35468747 PMC9037056

[16] Yeh SH Lin LW Chuang YK Liu CL Tsai LJ Tsuei FS. Effects of music aerobic exercise on depression and brain-derived neurotrophic factor levels in community dwelling women. Biomed Res Int. (2015) 2015:1–10. doi: 10.1155/2015/135893, PMID: 26075212 PMC4446469

[17] Jang DK Nam HS Park M Kim YH. Differences in associated factors of sedentary behavior by diabetes mellitus status: a nationwide cross-sectional study. J Clin Med. (2023) 12:5453. doi: 10.3390/jcm12175453, PMID: 37685520 PMC10487791

[18] Abdelbasset WK. Stay home: role of physical exercise training in older adults individuals’ ability to face the Covid-19 infection. J Immunology Res Hindawi Limited. (2020) 2020:1–5. doi: 10.1155/2020/8375096, PMID: 33354578 PMC7737455

[19] Cunningham C O' Sullivan R. Why physical activity matters for older adults in a time of pandemic. Eur Rev Aging Phys Act. (2020) 17:16. doi: 10.1186/s11556-020-00249-3, PMID: 32983273 PMC7509818

[20] Damiot A Pinto AJ Turner JE Gualano B. Immunological implications of physical inactivity among older adults during the COVID-19 pandemic. Gerontology. (2020) 66:431–8. doi: 10.1159/000509216, PMID: 32585674 PMC7362590

[21] Martin CR Preedy VR Rajendram R editors. Assessments, treatments and modeling in aging and neurological disease: The neuroscience of aging. Academic Press; (2021).

[22] Ramos AM Marcos-Pardo PJ Gomes R. Resistance circuit training or walking training: which program improves muscle strength and functional autonomy more in older women? Int J Environ Res Public Health. (2022) 19:828. doi: 10.3390/ijerph19148828, PMID: 35886680 PMC9319797

[23] Said CM Batchelor F Duque G. Physical Activity and exercise for older people during and after the coronavirus disease 2019 pandemic: a path to recovery. J American Medical Directors Association Elsevier Inc. (2020) 21:977–9. doi: 10.1016/j.jamda.2020.06.001, PMID: 32674830 PMC7269946

[24] Hamilton M. A rating scale for depression. J Neurol. Neurosurg. Psychiatry. (1960) 23:56–62. doi: 10.1136/jnnp.23.1.56 14399272 PMC495331

[25] Trajković G Starčević V Latas M Leštarević M Ille T Bukumirić Z. Reliability of the hamilton rating scale for depression: a meta-analysis over a period of 49 years. Psychiatry Res. (2011) 189:1–9. doi: 10.1016/j.psychres.2010.12.007, PMID: 21276619

[26] Brown LJ Astell AJ. Assessing mood in older adults: a conceptual review of methods and approaches. Int Psychogeriatr. (2012) 24:1197–206. doi: 10.1017/S1041610212000075, PMID: 22340813

[27] Yesavage JA Brink TL Rose TL Lum O Huang V Adey M. Development and validation of a geriatric depression screening scale: a preliminary report. J Psychiatr Res. (1982) 17:37–49. doi: 10.1016/0022-3956(82)90033-4, PMID: 7183759

[28] Lopez AO Martinez MN Garcia JM Kunik ME Medina LD. Self-report depression screening measures for older Hispanic/Latin American adults: A PRISMA systematic review. J Affect Disord. (2021) 294:1–9. doi: 10.1016/j.jad.2021.06.049, PMID: 34252863 PMC8410643

[29] Espino DV Lichtenstein MJ Palmer RF Hazuda HP. Evaluation of the mini-mental state examination's internal consistency in a community-based sample of Mexican-American and European-American elders: Results from the San Antonio Longitudinal Study of Aging. J Am Geriatr Soc. (2004) 52:822–7. doi: 10.1111/j.1532-5415.2004.52226.x, PMID: 15086669

[30] INGER. (2020). “Guía de Instrumentos de Evaluación Geriátrica Integral.” Available at: http://www.geriatria.salud.gob.mx/contenidos/institucional/publicaciones.html.

[31] Connor KM Davidson JRT. Development of a new resilience scale: the Connor-Davidson Resilience Scale (CD-RISC). Depress Anxiety. (2003) 18:76–82. doi: 10.1002/da.10113, PMID: 12964174

[32] Borrega-Mouquinho Y Sánchez-Gómez J Fuentes-García JP Collado-Mateo D Villafaina S. Effects of high-intensity interval training and moderate-intensity training on stress, depression, anxiety, and resilience in healthy adults during coronavirus disease 2019 confinement: a randomized controlled trial. Front Psychol. (2021) 12:3069. doi: 10.3389/fpsyg.2021.643069 PMC7943442 33716913

[33] Crespo MEO Lansac VF Soberón C. Adaptación española de la Escala de resiliencia de Connor-Davidson (CD-RISC) en situaciones de estrés crónico. Psicología Conductual = Behavioral Psychology: Revista Internacional De Psicología Clínica Y De La Salud. (2014) 22:219–38.

[34] Serrano-Parra MD Garrido-Abejar M Notario-Pacheco B Bartolomé-Gutierrez R Solera-Martínez M Martínez-Vizcaino V. Validez de la escala de Resiliencia de Connor-Davidson (CD-RISC) en una población de mayores entre 60 y 75 años. Int J psycholog res. (2012) 5:49–57. doi: 10.21500/20112084.736

[35] Jones CJ Rikli RE. Measuring functional. J Active Aging. (2002) 1:24–30.

[36] Aksay E. Live online exercise programs during the Covid-19 pandemic–are they useful for older adults adults. J Physical Educ Sport. (2021) 21:1650–8. doi: 10.7752/jpes.2021.04209

[37] Peyrusqué E Granet J Pageaux B Buckinx F Aubertin-Leheudre M. Assessing physical performance in older adults during isolation or lockdown periods: web-based video conferencing as a solution. J Nutr Health Aging. (2022) 26:52–6. doi: 10.1007/s12603-021-1699-y 35067703 PMC8590923

[38] Zengin Alpozgen A Kardes K Acikbas E Demirhan F Sagir K Avcil E. The effectiveness of synchronous tele-exercise to maintain the physical fitness, quality of life, and mood of older people-a randomized and controlled study. European Geriatric Med. (2022) 13:1177–85. doi: 10.1007/s41999-022-00672-y, PMID: 35881310 PMC9315330

[39] Middleton A Fritz SL Lusardi M. Walking speed: the functional vital sign. J Aging Phys Act. (2015) 23:314–22. doi: 10.1123/japa.2013-0236, PMID: 24812254 PMC4254896

[40] Peters DM Fritz SL Krotish DE. Assessing the reliability and validity of a shorter walk test compared with the 10-meter walk test for measurements of gait speed in healthy, older adults. J Geriatr Phys Ther. (2013) 36:24–30. doi: 10.1519/JPT.0b013e318248e20d, PMID: 22415358

[41] Kon SS Patel MS Canavan JL Clark AL Jones SE Nolan CM. Reliability and validity of 4-meter gait speed in COPD. Eur Respir J. (2013) 42:333–340. doi: 10.1183/09031936.00162712 23222875

[42] Krumpoch S Lindemann U Rappl A Becker C Sieber CC Freiberger E. The effect of different test protocols and walking distances on gait speed in older persons. Aging Clin Exp Res. (2021) 33:141–6. doi: 10.1007/s40520-020-01703-z, PMID: 32930990 PMC7897617

[43] De Abajo S Larriba R Márquez S. Validity and reliability of the Yale physical activity survey in Spanish elderly. J Sports Med Phys Fitness. (2001) 41:479–85. PMID: 11687767

[44] Borg GAV. Psychophysical bases of perceived exertion. Med Sci Sports Exerc. (1982) 14:377–81. 7154893

[45] Little RJ. A test of missing completely at random for multivariate data with missing values. J Am Stat Assoc. (1988) 83:1198–202. doi: 10.1080/01621459.1988.10478722, PMID: 38375077

[46] Dziura J Post LA Zhao Q Fu Z Peduzzi P. Strategies for dealing with missing data in clinical trials: from design to analysis. PubMed. (2013). Available at: https://pubmed.ncbi.nlm.nih.gov/24058309 PMC3767219 24058309

[47] Cohen J. A Power Primer. Psychol Bull. (1992) 112:155–9. doi: 10.1037/0033-2909.112.1.155, PMID: 19565683

[48] Salguero A Martínez-García R Molinero O Márquez S. Physical activity, quality of life and symptoms of depression in community-dwelling and Institutionalized older adults. Arch Gerontol Geriatr. (2011) 53:152–7. doi: 10.1016/j.archger.2010.10.005, PMID: 21035206

[49] Zenebe Y Akele B Selassie M Necho M. Prevalence and determinants of depression among old age: a systematic review and meta-analysis. Ann General Psychiatry. (2021) 20:55. doi: 10.1186/s12991-021-00375-x, PMID: 34922595 PMC8684627

[50] Dean J Keshavan M. The neurobiology of depression: an integrated view. Asian J Psychiatry Elsevier BV. (2017) 27:101–11. doi: 10.1016/j.ajp.2017.01.025, PMID: 28558878

[51] Hu S Tucker L Chongyun W Yang L. Beneficial effects of exercise on depression and anxiety during the Covid-19 pandemic: a narrative review. Front Psych. (2020) 11:587557. doi: 10.3389/fpsyt.2020.587557, PMID: 33329133 PMC7671962

[52] Rush AJ South C Jain S Agha R Zhang M Shrestha S. Clinically significant changes in the 17-and 6-Item Hamilton Rating Scales for Depression: a STAR* D report. Neuropsychiatr Dis Treat. (2021) 17:2333–45. doi: 10.2147/NDT.S305331, PMID: 34295161 PMC8290193

[53] Gourgouvelis J Yielder P Clarke ST Behbahani H Murphy BA. Exercise leads to better clinical outcomes in those receiving medication plus cognitive behavioral therapy for major depressive disorder. Front Psych. (2018) 9:37. doi: 10.3389/fpsyt.2018.00037 PMC5845641 29559928

[54] Cooney GM Dwan K Greig CA Lawlor DA Rimer J Waugh FR. Exercise for depression: some benefits but better trials are needed. Saudi Medical J. (2013) 34:1203. doi: 10.1002/14651858.CD004366.pub6

[55] Schuch FB Deslandes AC Stubbs B Gosmann NP Belem CT. Neurobiological effects of exercise on major depressive disorder: a systematic review. Neurosci Biobehav Rev. (2016) 61:1–11. doi: 10.1016/j.neubiorev.2015.11.012, PMID: 26657969

[56] Feder A Nestler EJ Charney DS. Psychobiology and molecular genetics of resilience. Nat Rev Neurosci. (2009) 10:446–57. doi: 10.1038/nrn2649, PMID: 19455174 PMC2833107

[57] Russo SJ Murrough JW Han MH Charney DS Nestler EJ. Neurobiology of resilience. Nat Neurosci. (2012) 15:1475–84. doi: 10.1038/nn.3234, PMID: 23064380 PMC3580862

[58] Ryan M Ryznar R. The molecular basis of resilience: a narrative review. Front Psych. (2022) 13:856998. doi: 10.3389/fpsyt.2022.856998, PMID: 35599764 PMC9120427

[59] Franklin TB Saab BJ Mansuy IM. Neural mechanisms of stress resilience and vulnerability. Neuron. (2012) 75:747–61. doi: 10.1016/j.neuron.2012.08.016, PMID: 22958817

[60] Silverman MN Deuster PA. Biological mechanisms underlying the role of physical fitness in health and resilience. Interface Focus. (2014) 4:20140040. doi: 10.1098/rsfs.2014.0040, PMID: 25285199 PMC4142018

[61] Carriedo A Cecchini JA Fernández-Río J Méndez-Giménez A. Resilience and physical activity in people under home isolation due to COVID-19: a preliminary evaluation. Ment Health Phys Act. (2020) 19:361. doi: 10.1016/j.mhpa.2020.100361 PMC7530639 33024452

[62] Eöry A Békési D Eöry A Rózsa S. Physical exercise as a resilience factor to mitigate COVID-related allostatic overload. Psychother Psychosom. (2021) 90:200–6. doi: 10.1159/000514331, PMID: 33691321 PMC8678241

[63] Batista FS Gomes GA d O D'Elboux MJ Cintra FA Neri AL Guariento ME. Relação Entre Força Muscular de Membros Inferiores e Independência Funcional de Idosos Segundo Critérios de Fragilidade: Um Estudo Transversal. Med J. (2014) 132:282–9. doi: 10.1590/1516-3180.2014.1325669, PMID: 25054965 PMC10496748

[64] Radaelli R Taaffe DR Newton RU Galvão DA Lopez P. Exercise effects on muscle quality in older adults: a systematic review and meta-analysis. Sci Rep. (2021) 11:600. doi: 10.1038/s41598-021-00600-3, PMID: 34702909 PMC8548567

[65] Charette SL McEvoy L Pyka G Snow-Harter C Guido D Wiswell RA. Muscle hypertrophy response to resistance training in older women. J. Appl. Physiol. (1991) 70:1912–1916. 1864770 10.1152/jappl.1991.70.5.1912

[66] Cadore EL Pinto RS Lhullier FLR Correa CS Alberton CL Pinto SS. Physiological effects of concurrent training in older adults men. Int J Sports Med. (2010) 31:689–97. doi: 10.1055/s-0030-1261895, PMID: 20617484

[67] Briggs R Carey D Claffey P McNicholas T Donoghue O Kennelly SP. Do differences in spatiotemporal gait parameters predict the risk of developing depression in later life? J Am Geriatr Soc. (2019) 67:1050–6. doi: 10.1111/jgs.15783, PMID: 30723898

[68] Huang TY Liang CK Shen HC Chen HI Liao MC Chou MY. Gait speed rather than dynapenia is a simple indicator for complex care needs: a cross-sectional study using minimum data set. Sci Rep. (2017) 7:8418. doi: 10.1038/s41598-017-08791-4, PMID: 28827697 PMC5566363

[69] Li L Simonsick EM Ferrucci L Lin FR. Hearing loss and gait speed among older adults in the United States. Gait Posture. (2013) 38:25–9. doi: 10.1016/j.gaitpost.2012.10.006, PMID: 23177614 PMC3845825

[70] Barha CK Liu-Ambrose T. Exercise and the aging brain: considerations for sex differences. Brain Plasticity. (2018) 4:53–63. doi: 10.3233/BPL-180067, PMID: 30564546 PMC6296261

[71] Devries MC Jakobi JM. Importance of considering sex and gender in exercise and nutrition research. Appl Physiol Nutr Metab. (2021) 46:iii–vii. doi: 10.1139/apnm-2021-0298, PMID: 34125618

